# Advanced Theranostic Strategies for Viral Hepatitis Using Carbon Nanostructures

**DOI:** 10.3390/mi14061185

**Published:** 2023-06-01

**Authors:** Ahmad Gholami, Seyyed Mojtaba Mousavi, Reza Masoumzadeh, Mojtaba Binazadeh, Kamran Bagheri Lankarani, Navid Omidifar, Omid Arjmand, Wei-Hung Chiang, Mohsen Moghadami, Nelson Pynadathu Rumjit

**Affiliations:** 1 Biotechnology Research Center, Shiraz University of Medical Science, Shiraz 71439-14693, Iran; gholami@sums.ac.ir; 2 Pharmaceutical Sciences Research Center, Department of Pharmaceutical Biotechnology, Faculty of Pharmacy, Shiraz University of Medical Science, Shiraz 71439-14693, Iran; 3 Department of Chemical Engineering, National Taiwan University of Science and Technology, Taipei 10607, Taiwan; whchiang@mail.ntust.edu.tw; 4 Department of Medical, Shiraz University of Medical Sciences, Shiraz 71439-14693, Iran; reza368rm@gmail.com; 5 Department of Chemical Engineering, School of Chemical and Petroleum Engineering, Shiraz 71557-13876, Iran; binazadeh@shirazu.ac.ir; 6 Health Policy Research Center, Health Institute, Shiraz University of Medical Sciences, Shiraz 71439-14693, Iran; 7 Department of Pathology, Shiraz University of Medical Sciences, Shiraz 71439-14693, Iran; 8 Department of Chemical Engineering, South Tehran Branch, Islamic Azad University, Tehran 14687-63785, Iran; omid.arjemand@gmail.com; 9 Non-Communicable Diseases Research Center, Shiraz University of Medical Sciences, Shiraz 71439-14693, Iran; moghadami@sums.ac.ir; 10 Nanotechnology and Catalysis Research Centre (NANOCAT), Level 3, Block A, Institute for Advanced Studies (IAS), University of Malaya (UM), Kuala Lumpur 50603, Malaysia; nelsonpynadath@gmail.com

**Keywords:** graphene-based nanomaterials, biomedical, biosensors, drug delivery, cancer diagnosis

## Abstract

There are several treatment protocols for acute viral hepatitis, and it is critical to recognize acute hepatitis in its earliest stages. Public health measures to control these infections also rely on rapid and accurate diagnosis. The diagnosis of viral hepatitis remains expensive, and there is no adequate public health infrastructure, while the virus is not well-controlled. New methods for screening and detecting viral hepatitis through nanotechnology are being developed. Nanotechnology significantly reduces the cost of screening. In this review, the potential of three-dimensional-nanostructured carbon substances as promising materials due to fewer side effects, and the contribution of these particles to effective tissue transfer in the treatment and diagnosis of hepatitis due to the importance of rapid diagnosis for successful treatment, were extensively investigated. In recent years, three-dimensional carbon nanomaterials such as graphene oxide and nanotubes with special chemical, electrical, and optical properties have been used for the diagnosis and treatment of hepatitis due to their high potential. We expect that the future position of nanoparticles in the rapid diagnosis and treatment of viral hepatitis can be better determined.

## 1. Introduction

By 2021, 1.1 million people had died from hepatitis, and about 90% of patients were infected with hepatitis B (HBV) and hepatitis C (HCV). HCV and HBV lead to liver fibrosis and increase the chance of liver cell carcinoma [1,2,3,4,5,6]. About 40–80% of humans infected with viral hepatitis are asymptomatic and uninformed of their disease until they become chronic with symptomatic hepatitis. Developing countries have the highest incidence and mortality from the chronic liver disease [7,8,9]. With the rapid diagnosis of hepatitis, the prevalence of viral hepatitis can be significantly controlled and reduced. More accurate and faster diagnoses lead to a more effective remedy and reduced treatment costs [10,11]. Conventional methods of detecting hepatitis viruses include chemical processes [12], ELISA [13,14], and PCR [15,16]. However, these methods have several disadvantages: high cost, false positive and negative results, and the need for experienced people [17,18,19,20]. Some available techniques for detecting derivatives produced by viral hepatitis include IgM and IgG for HAV, anti-HBc antibodies, HBe antibody, anti-HBV DNA, HBeAg, HBsAg, HCV RNA, anti-HCV antibodies, HDV antigen, anti-HDV antibodies, anti-HEV antibodies, and HEV RNA. Various laboratory diagnostic methods used to diagnose viral diseases include electron microscopy, detection of viral proteins, culture using serology, cytology, virus isolation, enzymes, identification of viral genomes, and antibody detection. Molecular techniques are one of the most reliable and accurate tools for diagnosing viral diseases. Molecular methods based on nucleic acid amplification (with three signals, probe amplification, and target) identify viral hepatitis [1,21,22]. Recently, biosensors have been used for biological work. Biosensors have various detection mechanisms, including acoustic, magnetic, selective, thermal, optical, and electrochemical [23,24,25]. Single-layer graphene is a flat monolayer of carbon atoms tightly arranged in a two-dimensional honeycomb structure by sp2 hybridization [26]. In recent years, graphene has received increasing attention from scientists worldwide due to its excellent electrical, thermal, optical, and mechanical properties. Graphene sheets have been used as building blocks for fabricating 3D graphene constructs with defect-free, pristine graphene properties. This can be achieved using graphene sheets as building blocks and 2D graphene constructs based on graphene sheets [27].

The current treatment options for chronic hepatitis B (CHB) infection are limited and have several drawbacks. Interferon (IFN) therapy and nucleoside analogs (NAs) are the most common treatments, but NAs require long treatment cycles and have a high rebound rate after drug withdrawal [28]. Additionally, there is a high rate of clinical resistance, and only a few patients are eligible for IFN therapy [29]. The current drugs cannot enter the nucleus and clear the cccDNA, which is the primary cause of chronic viral hepatitis infections and recurrence in patients with a negative HBsAg result after drug withdrawal. Furthermore, the integration of the HBV and host cell genomes during viral replication is closely related to chronic infection and the occurrence of hepatoma [30]. Fortunately, recent research has shed light on the lifecycle of HBV, revealing several novel, promising drug targets. With further studies and analysis, better treatment options may become available for CHB patients.

New research on novel drugs and pharmaceutical preparations provides innovative strategies for determining effective treatment, designing drug combinations, addressing clinical issues such as chronic infection control, and working towards a functional cure for viral hepatitis. Many novel anti-hepatitis drugs have been developed based on the virus’s lifecycle and host immune mechanisms [31]. However, certain critical factors must be considered to ensure curative effects, such as biological stability and safety, effective drug delivery, and controlled release.

Researchers have developed nanoparticle delivery systems for traditional NAs to address these concerns, which reduce the dosage and toxicity and improve the therapeutic index. These systems have been beneficial in enhancing the antiviral effectiveness and facilitating the development of resistant strains through targeted drug delivery for novel anti-hepatitis drug targets, thereby halting the progression of the disease. The number of nanoparticle formulations for anti-HBV therapy has dramatically increased, and this review aims to summarize the characteristics of carbon nanostructures that are effective in treating various types of viral hepatitis.

On the other hand, there has been a notable increase in utilizing carbon nanostructures for simultaneously diagnosing and treating various types of viral infections in recent years. Specifically, in cases of viral hepatitis, carbon nanostructures have been successfully employed to enhance theranostics, offering exceptional precision and flexibility. This review also provides an overview of the possible applications of carbon nanostructures in diagnosing and treating various forms of viral hepatitis. We discuss the potential benefits of integrating carbon nanostructures and nanotheranostics to improve the diagnosis and treatment of this disease. Recent advancements and concerns in the field of carbon nanomaterials are also summarized. We describe potential markers for detecting viral hepatitis and emerging carbon nanostructure platforms.

Additionally, we highlight the therapeutic targets and drug delivery methods that utilize carbon nanostructures to simultaneously diagnose and treat diseases. Finally, we offer our perspectives on engineering a safe, efficient, and specific carbon nanostructure system for the theranostic approach to viral hepatitis. This review aims to showcase the technological advances and strategies used to design surface-engineered carbon nanostructures that can effectively diagnose and treat viral hepatitis.

Graphene oxide (GO) is a chemically modified graphene usually prepared by exfoliation of graphite and can be converted into chemically reduced graphene oxide (rGO) [32]. GO has a 2D structure, similar to graphene, but the single layer of carbon atoms is covalently functionalized with oxygen-containing groups (hydroxyl, epoxide, carbonyl, etc.) on the basal plane and edges introduced during chemical exfoliation of graphite flakes [33,34]. The facile synthetic route and pore morphology make 3D reduced graphene oxide (rGO) an essential candidate for the 3D graphene group. Reduction by thermal, chemical, hydrothermal, electrochemical, and photoinduced routes is one of the effective techniques for reducing 3D GO or GO suspensions with simultaneous 3D assembly [35,36]. Multi-walled nanotubes (MWNTs) and single-walled nanotubes (SWNTs) can be classified as CNTs. SWNTs are coiled tubes of single-layer graphene with end caps and a tube diameter of generally 1 nanometer and a length of 1 m; therefore, they are considered long 1D materials [23]. Three-dimensional nanomaterials, the last dimensional class of nanomaterials, have only internal nanoscale features, but no external nanoscale dimensions. The development and optimization of carbon-based 3D nanostructures are crucial because the properties of each building block can be significantly improved if a suitable nanostructure is chosen for each [37]. Even though carbon-based 3D nanostructures exhibit structural interconnectivity, they create hierarchical porous channels and possess increased electrical conductivity and better structural/mechanical stability [38,39].

Electrochemical biosensors containing nanostructures, including nanotubes, and graphene oxide (GO), have been used to detect hepatitis. Indeed, this approach has unique properties, such as cost-effectiveness, electrical conductivity, biocompatibility, and convenience, as a fundamentally promising method [40,41]. Electrochemical immunosensors have made great strides in different scientific fields, such as medicine, agriculture, diet safety, forensic analysis, environmental control, prohibition, drug production, quality monitoring, and management of epizootic illness. Various architectures and strategies have been proposed for electrochemical safety sensors, including magneto immunosensors, impedimetric immunosensors, capacitive, quantum dots (QDs)-labeled immunosensors, and enzyme-labeled immunosensors. Recently, scholars have shown high attachment to nanostructures, such as reduced GO (rGO), non-porous materials, and carbon nanotubes (CNTs), due to their excellent properties [27,39]. This review article overviews recent advances in standard methods for diagnosing and treating viral hepatitis. It is intended to provide a snapshot of the current state of affairs. It is also discussed that nanostructured 3D carbon materials, including graphene oxide, rGO, and carbon nanotubes, can provide excellent options for diagnosing and treating the various forms of HBV and HCV.

## 2. Viral Hepatitis

Hepatitis means liver inflammation, which can be limited or progress to cirrhosis, fibrosis, and cancer. Many factors affect the development of hepatitis, including toxins (alcohol, certain drugs), autoimmune diseases, viral hepatitis, and infections as the most prevalent reasons for hepatitis. Viral hepatitis is divided into five main groups, A, B, C, D, and E. The main concerns for these five types of viruses are the potential for outbreaks, the severity of the disease, and the resulting death [42]. HCV and HBV types are the most common causes of cirrhosis and liver cancer, leading to chronic liver disease [43]—contaminated water and food cause the transmission of hepatitis E and A viruses. Hepatitis B, C, and D viruses are mainly through parenteral contact with body fluids. The viruses are usually transmitted through sexual communication, invasive medical procedures using infected equipment, the information of HBV vertical transmission, and receiving infected blood or blood products [44]. 

### 2.1. Current Methods for Diagnosing Viral Hepatitis

Diagnosing and testing HCV and HBV infections are essential for treating, preventing, and caring for patients. Early detection of people with these viruses and providing the necessary treatment and care could prevent liver disease progression [45]. Testing increases the opportunity for communication-reduction interventions, such as HBV vaccination, preparation of prophylactic products (sterile needles and syringes), and counseling on hazardous behaviors [46]. Table 1 summarizes all current methods for the diagnosis of viral hepatitis.

### 2.2. HBV Testing

The HBsAg marker is used to diagnose HBC infection. Serological methods have been used to identify this marker, such as the chemiluminescence immunoassay (CLIA), electrochemiluminescence (ECL), and the enzyme immunoassay (EIA). Two methods are used for HBsAg confirmation. In the first method, we use a particular reagent containing anti-HBsAg. Another serological test has a similar sensitivity to the second method [47,48]. The most critical factor in choosing the appropriate serology assessment method is the test site’s features (infrastructure, storage costs, facilities, and staff skill level) and the ease of use. In addition to serological tests, the measurement of liver function and phasing of liver illness based on clinical evaluation or non-invasive tests and HBV DNA levels play an essential role in the remedy [49,50]. HBeAg detection can be used to assess HBV viral infection. When HBV DNA testing is unavailable, tenofovir fumarate is used prophylactically in pregnant women with high levels of viremia to inhibit vertical transmission [51]. The market’s HBsAg and HBeAg rapid diagnostic tests (RDTs) have not met the requirements for WHO eligibility [52,53].

### 2.3. HCV Testing

HCV antibodies are used using serological methods to detect HCV infection (previous or current infection). For a person who has recently been infected, the antibody does not present for 2–3 months, so antibody-based methods cannot detect the diagnostic marker [54]. Methods that directly detect the HCV antigen contribute to diagnosing the infection over this 2- to 3-month period. Once HCV exposure is identified, the HCV virus infection test detects HCV RNA using qualitative, quantitative NAT, or HCVcAg [18,55].

### 2.4. The Way to Transmit Hepatitis Virus (HBV, HCV, HDV) 

All kinds of viral hepatitis are inherited. Genetics is the easiest way to transmit the virus. The gastrointestinal tract is one of the transmission routes because it is open to the outside environment [56]. Suppose we take a closer look at the transmission route of these viruses through the gastrointestinal tract. In that case, we will find that HBV is transmitted through fluids infected with the HBV virus, for example, blood, saliva, vaginal discharge, semen, tears, sweat, breast milk (infected mother during breastfeeding), and menstrual blood. An infected mother can also infect her baby during childbirth. Additionally, sexual intercourse is an essential factor (in the US, 65% of acute HBV cases are caused by sexual contact), as well as sharing syringes, tattooing, body piercing, and blood transfusion [57,58]. Although there are many transmission cases, the risk of contracting the virus is low. It is essential to note that HBV in the body does not mean the individual is infected. Many people are simply carriers of the virus. Some countries have screened for the virus for blood transfusions since 1975 [59].

The main route of transmission of the HCV virus is blood. In this way, the infected person’s bloodstream enters another person’s bloodstream, and from that moment on, the person will be infected [60]. Some HCV transmission cases include tattoos, body piercings, and medical procedures such as intravenous injections and blood transfusions (before 1990). However, unlike HBV, sexual contact and transmission during childbirth are ineffective routes of HCV [61].

The ways of transmission of HDV resemble those of HBV. The critical point is that the hepatitis D virus can only infect people who already have HBV; simultaneously, a person must be infected with the hepatitis B and D viruses. These cases are known as superinfections and are 70–95% more acute and chronic than hepatitis B. However, the body clears 90–95% of this infection (hepatitis D) [62,63].

Both HAV and HEV are transmitted through the gastrointestinal tract (feces). Poor health is one of the causes of the outbreak [64]. As a result, some countries with lower levels of health, such as India, South and Central America, and Bangladesh, present higher levels of HEV. Additionally, one-third of the US population is at risk of becoming infected with the hepatitis A virus [65].

### 2.5. Genetic Characteristics of Viral Hepatitis

Hepatitis A is a picornavirus that contains the single-stranded RNA genetic material with a positive 7478-base polarity. This sequence is encoded to produce polyproteins such as VP-3, VP-2, VP-1, and other viral proteins. HBV is one of the most critical diseases around the world. The virus’s genetic agent consists of 3226 bp organic organisms amplified by reverse transcription and expresses its gene through RNA-mediated transcription [66]. For surface proteins, nuclei, polymerases, and X have more than one shape code. Besides, different start codons are expressed differently, resulting in multiple products (proteins) [67]. The hepatitis C (non-A, non-B) genome comprises ssRNA of at least 10,000 bases that encode the antigen, specifically non-A and non-B. The hepatitis D virus is an infectious agent affecting the liver and contains an ssRNA genome with a ring of 1167 nucleotides. This genome encodes proteins p26 and P24, which bind to antiserum and are related to hepatitis D infection [68].

## 3. Carbon Nanoallotropes for Diagnosis and Treatment of Viral Hepatitis

Today, many carbon-based structures discovered in the past decades are studied and used in state-of-the-art devices to diagnose and treat various diseases. Nanodiamonds are carbon structures formed primarily by sp3 hybridization. The dopants in their structures have caused nanodiamonds to have unique electronic and optical properties. Additionally, the structural defects and unsaturated chemical bonds caused by carbon atoms in their chemical structures have caused the excellent surface activity of this category of carbon nanostructures.

Carbon nanostructures can be classified into two main groups based on the type of covalent bonds linking their C atoms. The first group comprises graphene nanostructures, predominantly sp2 carbon atoms tightly packed in a hexagonal honeycomb crystal lattice. This group includes graphene nanosheets, carbon nanotubes (CNTs), nano-horns, onion-like carbon nanospheres, and C-dots [69]. Graphene nanoallotropes are formed through the ability of carbon to form three identical covalent bonds with other carbon atoms using sp2 orbitals, producing a 2D lattice of hexagons. This group’s most straightforward and basic member is graphene—a thin, 2D sheet of sp2-hybridized carbon arranged in a hexagonal pattern. Graphene can form the building block for other graphic/graphitic nanoallotrope nostructures, such as nano-horns, obtained by elaborating on the structure of graphene in more sophisticated ways [27,34].

The second group of carbon nanostructures is composed of sp3 and sp2 carbon atoms in varying ratios and has mixtures of amorphous and graphitic regions or predominantly sp3 carbon atoms. Currently, nanodiamond is the only known member of this group, although some types of C-dots with non-graphitic structures could also be considered members. These nanoforms are not constructed from graphene parts or monolayers such as CNTs or single-walled carbon nano-horns (SWNHs).

Carbon nanoallotropes can be classified based on their morphological characteristics. The first category comprises nanostructures with empty internal spaces, including fullerene, carbon nanotubes, and nano-horns. These hollow structures can accommodate guest molecules, metals, atoms, or other nanostructures, and provide nano-environments that facilitate specific reactions. The second category includes robust nanostructures with no internal spaces, such as nanodiamonds, C-dots, and onion-like carbon (OLC) spheres. Graphene can also be classified under this category since it has no internal spaces. Another approach to classification is based on dimensionality, distinguishing between 0D structures such as fullerenes, OLC structures, C-dots, and nanodiamonds, 1D nanoallotropes such as CNTs, carbon nanofibers, and SWNHs (although the latter are organized into 3D aggregates), and 2D nanoallotropes such as graphene, graphene nanoribbons, and few-layer graphene.

**Figure 1:**
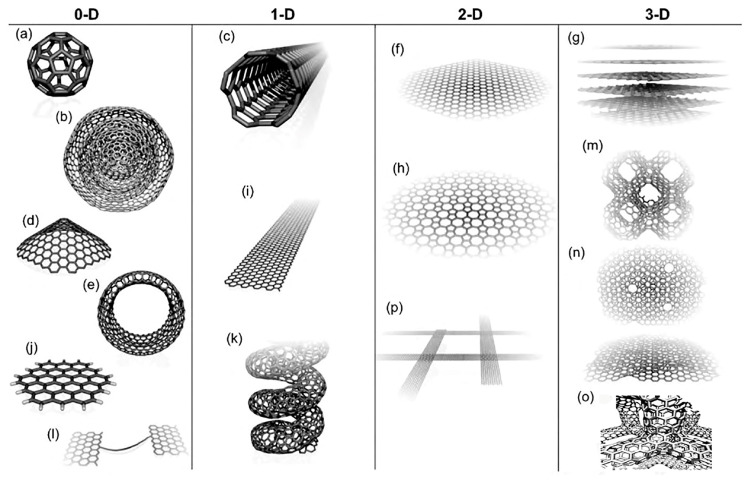
Molecular models of different types of sp2-like hybridized carbon nanostructures exhibiting different dimensionalities, 0D, 1D, 2D, and 3D: (**a**) C60: Buckminsterfullerene; (**b**) nested giant fullerenes or graphitic onions; (**c**) carbon nanotube; (**d**) nano-cones or nano-horns; (**e**) nanotoroids; (**f**) graphene surface; (**g**) 3D graphite crystal; (**h**) Haeckelite surface; (**i**) graphene nanoribbons; (**j**) graphene clusters; (**k**) helicoidal carbon nanotube; (**l**) short carbon chains; (**m**) 3D Schwarzite crystals; (**n**) carbon nanofoams (interconnected graphene surfaces with channels); (**o**) 3D nanotube covalent network following a tetragonal (or diamond-like) array; (**p**) nanoribbon 2D networks [70,71].

The molecular allotrope of carbon that consists of a three-dimensional closed cage (Cn) is called fullerene, which consists of five- and six-membered rings with twelve pentagons and different hexagons depending on the fullerene size. Carbon nanotubes are more complex structures of the fullerene family that form a quasi-one-dimensional structure (single- or multi-walled carbon nanotubes). The first two-dimensional atomic crystal discovered is graphene, formed by a single atomic layer of carbon atoms arranged in a honeycomb lattice structure. Figure 1 shows some types of carbon-based nanostructures along with their dimensions. Continuing this review article, we will discuss two critical categories of nanostructures used in 3D devices with applications in the treatment or diagnosis (or both simultaneously) of viral hepatitis.

## 4. Graphene Oxide

Chemically investigated oxygen content, good biocompatibility, better conductance, and extensive levels, as essential and excellent chemical properties, made GO a promising material for particular applications [34,70]. Functional groups, including epoxy, carboxyl, and hydroxyl, make graphene oxide easily able to be coordinated with other materials or molecules [38,71]. GO has hexagonal arrays, in which an sp-bond joins thick sheets of carbon atoms. GO can be made from a highly oxidized form with the help of potent oxidizing agents [37,72]. Besides, GO is chemically exfoliated from graphene derivatives due to its fluorescence cooling ability, water appearance, amphiphilicity, excellent surface performance ability, and Raman surface dispersion property [27,37,38]. In GO, the carbon layers are dispersed and reduced by oxygen molecules; eventually, the carbon layers are converted to multilayer or single-layer graphene. One of the by-products of this oxidation is graphene oxide because, in the reaction of graphite with oxidizing agents, the distance between the graphite layer plates increases (Figure 1) [27]. GO is produced by dispersing a fully oxidized compound in a base solution such as water.

Both sides of a graphene sheet contain many oxygen functional groups [73,74]. The presence of functional groups increases the distance between the layers by overcoming van der Waal’s interlayer force [26]. However, graphene oxide sheets have several defects that may result from oxidation and the amount of oxidant added. Graphene oxide was produced by Hummers, Brodie, and Staudenmeir [75]. The reduction of graphene oxide is significant for producing reduced graphene oxide (rGO) because it dramatically impacts the quality of rGO produced and determines how structurally close rGO is to pristine graphene (Figure 1). 

Graphene and its derivatives, such as those functionalized with polymeric molecules, have found wide biomedical applications. Table 2 summarizes the biomedical applications of these valuable nanostructures and the consequences of their use, along with the polymer molecules used.

### 4.1. Detection of Hepatitis Virus Antigens with Graphene Oxide

Electrochemical sensors have accurate, fast, and sensitive measurements for HBsAg detection. Unlabeled amperometric safety sensors are the best electrochemical safety sensors to detect HBsAg [82,83]. Choosing reliable modifiers is very important for providing excellent safety sensors. Graphene and graphene oxide have many biomedical applications, and one of the most significant biosensors based on graphene oxide is considered and utilized [26,75]. GO has advantages such as vast external areas, supreme film formation, better hydrophilicity, and easy preparation [84]. Using Raman surface spectroscopy (SERS), a combination of gold nanorods (GO-GNRs) and graphene oxide is designed to detect HBsAg [85]. Owing to the attendance of a vast number of carboxy and hydroxy groups at the GO surface, it acts as a substrate for the decoration of GNRs. Additionally, it immobilizes antibodies against HBsAg [85]. SERS activity in GNR is high due to the SERS 2-mercaptopyridine probe carrier’s presence, so biosensors are very sensitive. Antibodies at the GO-GNR level and HBsAg bind with high specificity and selectivity [85,86].

Serum HBsAg was measured in 4 female and 5 male patients (9 patients) to apply GO-GNR Raman Tags. Then, 1.00 mL of serum from the patient was diluted by 0.1 M phosphate buffer to the pg∙mL^−1^ level of HBsAg density. The serum HBsAg of the patient’s serum was experimentally measured, and the recovery was determined by adding different doses of antigen to the sample matrices. Accuracy assessment was calculated with RSD, and the spike recovery test determined the reliability of this method. The diagnosis of HBsAg with the Raman immunoassay is highly accurate because the recovery was 96–104%, and the RSD was less than ±5%. Therefore, to diagnose HBV infection and measure HBsAg in patients’ serum, the GO-GNRs Raman immunoassay is a clinically effective method [85].

Multilayer GO/Fc-CS/Au NP films are a new unlabeled electrochemical immunosensor method for detecting HBsAg. Au and GO/Fc-CS NPs used the Layer-by-Layer (LbL) adsorption method to form a multilayer film and were alternately modified in GE. Eventually, these multilayer films led to the formation of antibody binding sites and reversible redox signals. Fc-CS is an intermediary to enhance the electron conductivity of GO, guarantee the stability of bioactivity, and create a reversible redox signal. Another layer of film is Au NPs, that maintain good biocompatibility, antibody binding sites, and further strengthening of electron transfer for GO [84].

### 4.2. Treatment of Hepatitis with GO (HCV, HBV)

Numerous studies have been performed to detect viral enzyme inhibitors, particularly non-structural protein-3 serine protease, and NS5B-RdRp, identifying direct-impact antiviral factors for the remedy of HCV. ssDNA is converted from dsDNA by the C-terminal two-thirds of HCV-NS3, making a helicase [87,88]. One of the most critical HCV enzymes is NS3 helicase, used with NS5B RdRp. Five active inhibitors against helicases and five specific ones for each were identified from the primary and secondary screens, and 50–500 μM inhibitors displayed IC50 values in the target helicases [89]. Additionally, the target helicase was directly inhibited by ATPase. Among the five combinations known as HCV-NS3 helicase repressors, combination two inhibits HCV gene replication in human Huh-7 hepatocytes. The mGOHA screening method has a high throughput without false positive production, offering strong antiviral therapy [90]. This new method also provides real-time quantitative monitoring of helicase activity, providing reliability and reproducibility and overcoming the limitations of existing processes [91]. 

Current treatments for HBV include IFN (Pegasys), interferon (IFN)-α, adefovir (Hepsera), lamivudine (Epivir), entecavir (Baraclude), tenofovir (Viread), and telbivudine (Tyzeka) [92]. Treatment for HBV has limitations, such as adverse side effects, high costs, drug resistance, and liver failure risk during liver flares. New studies are looking for new therapies using nanotechnology to treat HBV. Wang et al. conducted an in vitro study and prepared different cationic nanoparticles of degradable polymers using nano-deposition and solvent evaporation methods [93]. To produce hepatitis B surface antigen (HBsAg), these nanoparticles’ transfection efficiencies in DNA delivery and siRNAs were evaluated. Various studies have shown that GO’s polyethyleneimine (PEI) layer has the highest anti-HBV effect. Successful siRNA delivery depends on the surface load and size.

## 5. Carbone Nanotubes (CNTs)

CNTs are used in safety sensors due to their unique properties among many nanomaterials. The considerable surface of CNTs is one of the unique features that allow them, when connected to high-density proteins, to act as matrices and as good candidates for safety sensors [23]. They are also appropriate for electrochemical safety measurements due to their excellent electrical conductance [94]. Since the discovery of CNTs in 1991, they have made great strides in most engineering and science fields owing to their extraordinary physical and chemical attributes. CNTs are among the best candidates for progressive amplifier substances in compounds due to their thermal, electronic, and mechanical properties [95].

**Figure 2:**
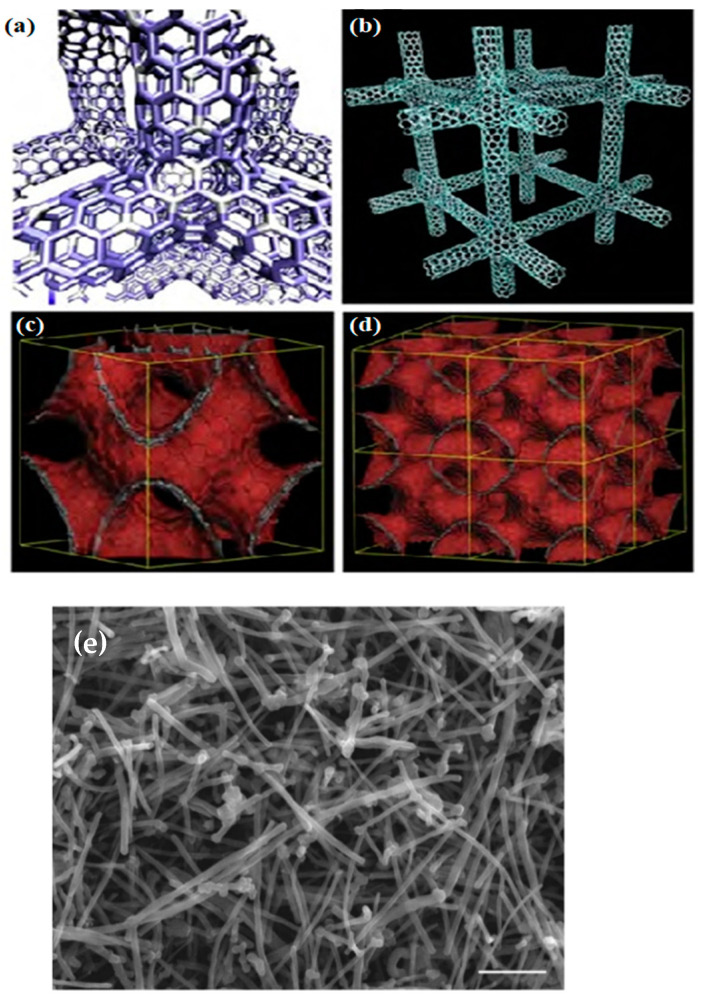
Molecular models of various carbon nanotube and graphene 3D networks containing hexagonal, heptagonal, pentagonal, and octagonal carbon rings: (**a**) Covalent carbon nanotube 3D network following a tetragonal (or rhombic) arrangement. (**b**) Cubic covalent carbon nanotube 3D network. (**c**,**d**) Periodic negatively curved graphene 3D structures (or foams) generated by covalently connecting graphene fragments with non-hexagonal rings [71]. (**e**) Scanning electron microscopy (SEM) image of MWC [100].

CNTs are empty carbon combinations with nanometer-scale diameters, one to several walls, and relatively more critical lengths. Carbon atoms regularly combine through sp2 bonds to form the most robust and rigid fibers (Figure 2) [39,96]. Compared to other NMs, used CNTs have an incomparable composition of electrical, chemical, magnetic, optical, and mechanical properties appropriate for vast usages, such as in biosensing [23]. Besides, CNTs have two functions: hexahedral functionalization (acting as platforms for combining other compounds on the surface) and endohedral functionalization (opening and filling without losing their stability) [96]. Functional CNTs quickly pass biological obstacles, such as cell membranes, and interpenetrate single cells. Biological synthesis programs, mainly intracellular, pay the most attention to this property, as well as diffusion of CNTs by cells and internalization [97,98,99].

CNTs have recently found many medical applications, as with proper modification, they can produce diverse systems, including protein transport systems or vaccine delivery systems (Table 3) [101], which will be described below.

CNT-based biosensors: CNTs have interesting electrical and electrochemical properties, making them ideal as electrode and electrode-biosensor converter components. SWCNTs are very sensitive to their environment. Chemical-sensitive anti-chemical transistors and chemical transistors detect biomolecules [103].

Hyperthermia therapy: A variety of nanomaterials, including multi-walled and single-walled carbon nanotubes, are produced in thermal tumor ablation treatments. Carbon nanotubes (CNTs) are very useful due to their simultaneous treatment and imaging potential [23,104].

Magnetic resonance imaging (MRI): MRI is one of the most widely used tomographic methods due to its ability to image soft, non-invasive tissue and its high spatial resolution. Carbon nanotubes provide an excellent scaffold for contrast media. The morphology of MWCNT, its electronic and magnetic properties, the possibility of penetration into the cell membrane, and the potential of different functions lead to a very attractive and unique candidacy for the new MRI contrast agent [105].

Drug delivery: Developing new and efficient drug systems is essential in increasing the drug profile of many molecular therapeutic groups. CNTs can be functionalized with drugs, nucleic acids, proteins, and biologically active peptides, and their shipments can be targeted to organs and cells. CNTs have great potential in functional pharmaceutical medicine and nanotechnology because they are not immunogenic and show little toxicity.

### 5.1. Detection of HBV Antigens with CNT

Much research has been carried out on electrochemical measurements based on carbon nanotubes (CNT) for detecting HBV antigens. Iijima reported several properties of CNTs with the rediscovery in 1991. CNTs have special features, such as fast electron transfer kinetics and significant changes in conductance, that make these materials suitable for Faradaic and non-Faradaic processes, respectively [106]. 

**Figure 3:**
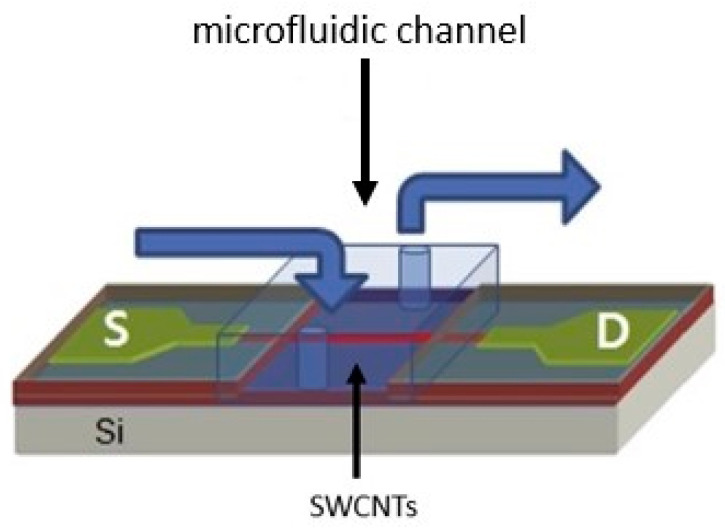
Schematic of the SWCNT biosensor with the FET structure. The microfluidic channel was mounted on the SWCNT biosensor for detecting hepatitis B [107]. In this image, the letter S represents the sample in which the virus is supposed to be detected, and the letter D represents the detector.

As shown in Figure 3, the CNT biosensor has a FET structure. Jeseung Oh et al. grew SWNT using a model catalyst growth method. Fe/Mo was modeled on heavy-doped Si layers by a thermally grown SiO2 layer with a width of 200 nm using alumina catalysts. Then, SWNTs were developed at 900 °C using CVD in a combination of argon and methane (for carbon provenance). As reported, a polydimethylsiloxane (PDMS) microfluidic channel is used for real-time detection on the device [107].

CNT is first incubated in a 1 mM N-hydroxyl succinimide acid methanol solution to produce the active amino succinimide ester. Then, the pyrene remainder is attached to the SWNT’s lateral walls [108]. HBV antibodies are injected as a solution on the CNT surface and immobilized for 3 h. Simultaneously adding HBV antigen to the microfluidic channel, electrical conductivity (as a function of time) is measured to detect HBV. Microfluidic channels are the site of all binding events (between antibodies and antigens) and chemical changes [107]. 

### 5.2. Treatment of Hepatitis with CNT

Thanks to their distinctive chemical and physical attributes, CNTs have many industrial and commercial sector applications [109]. These carbonaceous nanostructures are carbon grids with outstanding thermal and electrical conductivity and excellent nanometer-scale tensile stability. The discovery of these biotechnological materials allows access to the depths of previously inaccessible target organs and cells because CNTs can easily cross cell membranes. Therefore, traditional drug delivery methods are not more effective in delivering vaccines, biomolecules, genes, and drugs. Hence, nanomaterials can profoundly change therapeutic implications in the future and increase the likelihood of treating many incurable diseases [110].

**Figure 4:**
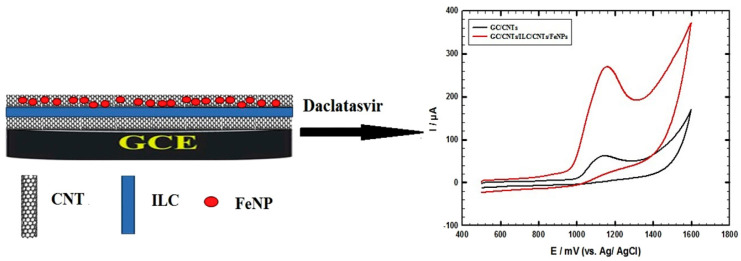
The modified electrode, made of MWCNTs and GC/ILC/FeNPs, is used for the electrochemical oxidation of DAC [107].

**Figure 5:**
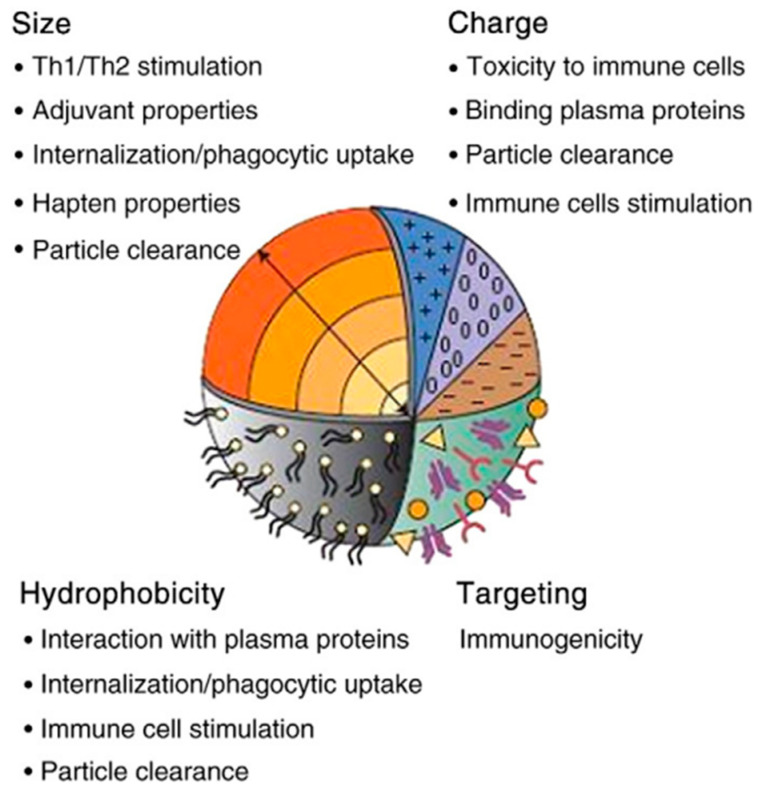
Important factors involved in the biocompatibility and toxicity of three carbon-based nanostructures [113].

Several drugs showed 95% successful HCV treatment, including daclatasvir and velpatasvir (Figure 4). Nanotechnology can be used for 5% drug resistance. For the treatment of patients with HCV, direct-acting antiviral drugs (DAAs) can play an essential role, as proven, and determining the level of daclatasvir (DAC) from the class of DAAs (recently introduced) is attributed to a straightforward and effective method of treatment [111]. As shown in Figure 5, a slim ILC layer is placed among two layers of MWNTs in the conductive composite design, with a layer of nanomagnetic iron oxide (CNTs/ILC/CNTs/FeNPs) [112]. The presence of ILC creates a compelling interplay with CNTs, subsequently providing a very ionic electronic compound with a large surface area for the assembly of magnetite FeNPs. CNTs have a large surface area for metal deposition, leading to synergistic interactions for enhanced electron transfer. This combination is a direct sensor to characterize the DAC [112]. 

## 6. Biocompatibility of 3D Carbon Nanostructure in the Physiological Condition of the Body

As 3D carbon nanomaterials have found many usages in different technological and scientific fields, concerns about these substances’ biocompatibility, toxicity, and safety are increasing daily [38,113,114,115,116]. The biocompatibility and toxicity of 3D carbon nanomaterials depend on many factors, including the coating, crystallinity, oxidative stress functions, particle size, concentration, stimulating secretion of by-products, the degradation process, surface energy, roughness, and the inherited chemistry [37,117,118]. It is difficult to pinpoint each factor’s particular role in the biocompatibility and toxicity of nanomaterials, but the most critical factors are briefly discussed below (Figure 5).

Size: Nanomaterials quickly pass through cell membranes to reach various organs and blood. These materials have more chemical molecules on their surface due to their high surface-to-volume ratio (compared to homogeneous materials with larger sizes). Therefore, this could be one of the reasons NPs are more poisonous (compared to particles more significant than the same compound) [119,120,121,122].

Surface attributes and chemical composition: The chemical composition and chemicals adsorbed on the surface of these 3D nanomaterials determine the degree of toxicity. Therefore, we can minimize the damage by changing the surfaces of nanoparticles [123,124].

Shape: The shape of nanoparticles is one of the essential factors affecting health. For example, nanotubes are tiny in diameter (several nanometers) but have a length of several micrometers. The relationship between toxicity and shape is such that the toxicity is enhanced by altering the shape, from an equiaxed to an acicular shape [125,126,127,128].

## 7. Electrochemical Mechanism of 3D Carbon Nanostructure in Detecting Hepatitis Virus

To detect the presence of a virus, electrochemical biosensors are being developed to detect virus-specific molecules, such as nucleic acids, antigens, and antibodies [129]. It is possible to develop graphene-based electrochemical immunosensors based on the selective affinity of ssDNA for dsDNA on graphene. Non-covalent interactions between nucleobases and graphene surface lead to immobilizing single-stranded nucleic acids with exposed nucleobases on graphene [130]. In contrast, the rigid structure of dsDNA and the negatively charged backbone of dsDNA have a lower affinity for graphene. Biosensors have been constructed using graphene/GO to detect nucleic acids, proteins, and small molecules due to their better binding to ssDNA than dsDNA [131,132]. The interactions between graphene and RNA are similar, as RNA has a similar structure to DNA. However, the high susceptibility of RNA to degradation by enzymes makes it less commonly used in sensors. In addition, researchers) reported that GO ssRNA effectively protects against this enzymatic cleavage [133]. Nucleic acid probes used in DNA hybridization-based electrochemical sensing are designed to be highly selective and specific, such that their binding to complementary target nucleic acid strains allows identification of the target, even in the presence of a mixture of nucleic acids. While DNA-based analytical techniques provide precise, sensitive, qualitative, quantitative, and accurate target detection, they depend on temperature, pH, ionic strength, and the DNA concentration. As a result of an aptamer-based proximity binding strategy, an electrochemical aptasensor was developed to detect ultrasensitive viruses, including the hepatitis C (HCV) core antigen [133]. Short-sequence oligonucleotides (ssDNA) related to HCV can be rapidly and effectively detected using a label-free electrochemical biosensor. GO was incorporated into a pencil graphite electrode. The purine/pyrimidine rings of the ssDNA probe came into contact with the aromatic domains of GO, resulting in ssDNA interactions with the surface of GO. Hybridization of the ssDNA probe with the target DNA HCV generated dsDNA that readily desorbed from the surface of GO. As a result, the guanine oxidation signal obtained by DPV was directly affected.

In the range of 0.1 nM to 0.5 mM, the oxidation peak current was proportional to the concentration of the complementary strand concerning HCV, and the value of LOD was 4.3 × 10^−11^ M [131]. The hepatitis B virus core antigen (HBcAg) was immobilized on an AuNPs-decorated rGO nanocomposite (rGO-en-AuNPs) to serve as an antigen-functionalized surface and to detect the presence of anti-HBcAg. In the presence of anti-estradiol antibodies and bovine serum albumin as interferences, modified rGO-en-AuNPs/HBcAg were used for impedimetric detection of anti-HBcAg. Detection of anti-HBcAg was successful in both sample buffers spiked with human serum and serum spiked with buffer samples. Electron transfer resistance was linearly related to the concentration of anti-HBcAg, with LOD varying from 3.91 ng mL to 125.00 ng mL, the highest and lowest detection limits, respectively, depending on the concentration of anti-HBcAg [129]. Electrochemical biosensors that detect specific biomarkers enable high sensitivity and selectivity between viruses. Several biomarkers can be used for hepatitis diagnoses, such as hepatitis B surface antigen (HBsAg), hepatitis B surface antibody (Anti-HB), hepatitis B e-antigen (HBeAg), hepatitis core antigen (Anti-HBc), and HBV DNA. Quantifying biomarkers in body fluids also determines the level of HBV infection [132]. One of the foremost widespread concerns regarding using carbon nanostructure-based analytical systems as biomedical devices is their indigenous cell toxicity, which remains debated. The experimental works reveal contradictory outcomes. Some studies evaluated the effects and consequences of carbon-based nanostructures on human cell lines, such as hepatocarcinoma, epithelial adenocarcinoma, and human breast adenocarcinoma cells [38,134]. The biocompatibility of carbon-based nanostructures decreased from rGO to CNT, GO, and nanodiamonds. These structures have even more adverse effects on cell lines with a more rapid division, such as Caco-2. The central factors responsible for their serious complications, such as decreased cell viability, enhanced levels of radical oxygen species (ROS) production, and apoptosis/necrosis, were their hydrophobicity and morphological characteristics [134]. Conclusively, despite the multiple impediments that still need to prevail before using carbon nanostructures for theranostic applications (such as the long-term adverse effects), this review emphasizes that an evaluation and risk-to-benefit calculation is pivotal to developing advanced multifunctional carbon nanostructure-based analytical systems for the detection and treatment of viral hepatitis.

## 8. Engineered Carbon Nanostructures for Strain-Specific Hepatitis Detection

Many optical and electrochemical immunosensors based on carbon nanostructures have been developed due to their exceptional electronic and chemical properties and large surface areas. These nano-biosensors are highly sensitive and can precisely detect hepatitis antigens [135,136]. Table 4 summarizes some recently developed carbon nanostructure-based nano-biosensors for strain-specific viral detection. Immunosensors are typically created by fixing an antibody onto the electrode’s surface, which can then specifically bind to its corresponding antigen. To enhance biosensing devices’ sensitivity and response time, some researchers have explored using CNTs due to their exceptional mechanical and electronic properties [107]. Ma and colleagues have developed a method that utilizes CNTs as a nanocarrier and combines it with DNA hybridization chain reaction (HCR) to enhance the signal for detecting the hepatitis C virus (HCV) core antigen [137]. A modified electrode was created using graphitized mesoporous carbon-methylene blue composites.

Additionally, Au NPs were electrodeposited onto this electrode to immobilize the HCV core antibody (Ab1). The secondary antibody layer was formed by attaching a bridging DNA probe and a secondary antibody to MWCNTs-COOH. A biotin-tagged signal DNA probe and an auxiliary DNA probe were bound with BP through HCR. HRP, a redox enzyme, was linked to DNA probes through the biotin-streptavidin system (multi-sHRP-DNA-CMWNTs). The HCV core antibody (Ab2) was labeled on multi-sHRP-DNA-CMWNTs, enabling the immunosensor to obtain detection signals at low analyte concentrations. The proposed immunosensor showed an excellent linear relationship in the HCV core antigen concentration, ranging from 0.25 pg/mL to 300 pg/mL, with a low limit of detection (LOD) of 0.01 pg/mL under optimized conditions. This immunosensor provides a reliable method to obtain an ultra-low LOD through covalently engineered CNTs.

There have been proposals for electrochemical biosensors based on CNTs that can accurately detect specific antibodies of certain hepatitis viruses [108]. In their research study, Cabral et al. utilized a film made of hyaluronic acid and CNTs to bind proteins in an electrochemical immunosensor. This innovative method effectively detected antibodies to hepatitis B (anti-HBc) with specificity [108]. Another electrochemical biosensor that uses HA-CNTs has been developed. This biosensor allows for specific antigens to be immobilized on it, resulting in increased electron transfer and high diagnostic sensitivity. The linear range of the immunosensor is between 1 and 6 ng/mL, with a LOD of 0.03 ng/mL. This biosensor offers rapid analysis and does not require incubation with antibodies, making it a promising tool for specific anti-HBc assays.

Li et al. have introduced a new electrochemical DNA biosensor that can detect strain-specific hepatitis virus DNA without labels [147]. Using carbodiimide chemistry facilitated by EDC and NHS, the MWCNTs-COOH were tailored onto 4,4′-diaminoazobenzene (4,4′-DAAB)-functionalized glassy carbon electrodes. As a result, the electrochemical DNA biosensor exhibited outstanding chemical stability and specificity towards DNA sequences of HBV. The covalently modified CNTs are a proficient method for enhancing the performance of biosensors, enabling the sensitive detection of viruses.

## 9. Human Health Risks and Safety Pattern of Carbon Nanostructures

The growing interest in carbonaceous nanostructures has led to concerns about potential health risks for humans. Studies in recent years have focused on carbon nanotoxicity, particularly regarding multi-walled carbon nanotubes, which share some geometric similarities with asbestos fibers and are carcinogenic in mouse models [148,149]. These concerns have extended to encompass all types of carbon nanostructures, attracting attention from the media and regulatory agencies tasked with assessing new chemical substances’ health and environmental risks.

The International Agency for Research on Cancer (IARC) has officially classified MWCNT-7, a specific type of commercially available multi-walled carbon nanotubes, as “possibly carcinogenic to humans”. This classification is based on sufficient evidence from toxicological studies and emphasizes the need for the responsible introduction of such materials into the market to avoid significant human exposure. However, this finding does not necessarily indicate human health risks for other types of carbon nanostructures. The IARC working group recognizes that different types of carbon nanotubes cannot be classified at this time due to insufficient evidence, particularly concerning chronic endpoints. Therefore, researchers must continue identifying the underlying physicochemical properties that govern the biological response to all 1D carbon forms [150].

Recent studies have explored the impact of different types of carbon nanostructures and surface formulations on biological responses. Researchers have expressed concerns about the potential risks associated with long, rigid CNTs compared to more flexible or “tangled” CNTs, which tend to be thinner [149,151]. To mitigate these risks, some studies have investigated ways to make CNTs more hydrophilic, which reduces bundling and aggregation [152,153]. This, in turn, decreases the effective length of the aggregate structure and reduces the likelihood of pathogenicity or carcinogenicity [154,155]. For example, researchers have shown that coating CNTs with a non-ionic amphiphilic polymer called Pluronic can reduce their profibrogenic effects in vitro and in vivo [100]. In the biomedical field, toxicity data are commonly reported [156], and researchers frequently modify raw CNT characteristics through shortening and functionalization strategies to promote dispersion [157,158]. These treatments often produce short, hydrophilic varieties that do not pose pathogenicity risks at the doses applied. Additionally, published evidence suggests that N-doping may improve CNT biocompatibility [159,160].

The type of carbon nanostructures also has an impact on exposure. Nakanishi et al. conducted a study on six different types of nanotubes, considering occupational exposure and the biological response. They proposed a hazard quotient based on this information [161]. The study revealed significant variations among CNT types, with SWCNTs posing the lowest risk. It was suggested that workplace risks could be mitigated by reducing the concentration of airborne micron-scale aggregates (1 mm < d < 4 mm) containing most of the respirable mass.

Material science and particle technology may hold promise in effectively managing and reducing the potential health risks associated with carbon nanotubes and other types of carbon nanostructures [162]. As the industry shows increased interest in graphene and its related 2D carbon forms, it is crucial to consider the lessons learned from carbon nanotube research and apply them early [163,164]. In December 2014, the Scientific Committee on Emerging and Newly Identified Health Risks categorized graphene nanomaterials as hazardous, recommending an urgent risk assessment [165]. Properly assessing new materials’ safety profile and health impacts is crucial in translating research into any commercial application. While some graphene materials have been found to cause adverse biological responses [27,166], further research is needed to understand the behavior of the diverse range of 2D carbons with varying thicknesses, lateral dimensions, and surface chemistry. Conflicting data on graphene materials have also been reported in the literature [165], similar to the early findings on carbon nanotubes. It will take time and continued research to entirely understand how graphene behaves in living systems.

Further research is necessary to understand the biological responses to the carbon nanomaterial group, and it is essential to carefully document and consider their physicochemical properties and how they interact with biological systems. To effectively manage the potential health impacts of carbon-based nanomaterials, it is crucial to: (a) conduct comparative studies on well-defined material libraries with systematic variations in physicochemical properties, (b) measure responses at various scales, such as cells, organs, and tissues, (c) benchmark results against previous studies to learn from past experiences, and (d) establish necessary constraints on formulation, handling, and use to manage exposure, as proposed for specific nanotubes. By conducting a robust program of research, comparative analysis, and regulation, we can promote safe manufacturing, development, and use of nanomaterials, assess technological risks against risk perception, and develop new forms of ethics for societal acceptance of novel technologies based on nanomaterials.

## 10. Conclusions

Considering that the treatments used for patients to date have not been sufficiently effective and safe, many people have complained about the harmful side effects of the drugs or their negligible impacts on the course of their disease. In hepatitis patients, nanotechnology could play a critical role in transmitting imaging and therapeutic compounds. Several 3D carbon nanomaterials under clinical or commercial testing are reportedly used in the imaging and treatment of liver disease. A series of 3D carbon nanostructured materials have been introduced to reach practical clinical efficacy, and their capabilities for clinical application have been substantially considered. To this end, aiming to achieve a targeted treatment and reduce current limitations in diagnosing and treating liver fibrosis, 3D carbon nanostructured materials with a unique feature could significantly improve the treatment indexes, as expected. It is suggested that for an effective and targeted clinical outcome, 3D carbon nanostructured materials should be chemically modified and enhanced by particular ligands, and the natural ligands could provide a highly effective performance concerning the targeted site. It is concluded that nanotechnology is a multidisciplinary scientific field with significant advances. Revolutionary advances in medicine can be expected with the advent of nanotechnology. Therefore, further experimental and clinical investigations are urgently needed and must be seriously considered.

## Figures and Tables

**Table 1:** Comparison of standard methods of diagnosing viral hepatitis [17].

Method	Basis of Action	Advantage	Disadvantage
Enzyme-linked immunosorbent assay (ELISA) and enzyme immunoassay (EIA)	Formation of antigen and antibody complexes	Performing a wide range of experiments, the ability to perform experiments by automation, serving a large number of tests in one shift with a small number of experts. Laboratory, long shelf-life with a proper expiration date of kits, no production of hazardous materials, such as radioactive materials, suitable and desirable sensitivity, and properties.	Low sensitivity in recognizing biomolecular materials such as microRNAs and a laborious/tedious assay system.
Dot blot assay	Identification of nucleic acids and proteins	High specificity, identify false positives, ability to detect antibodies against any of the virus antigens.	Relatively expensive, it is not performed as the first test and is mainly used to confirm the positive results of the ELISA test.
Chemical processes		Low cost, rapid, simple, higher sensitivity, selectivity, accurate, reproducibility.	-
Polymerase chain reaction (PCR)	Qualitative and quantitative evaluation of hepatitis virus RNA	The best diagnostic method in people with suppressed immune systems. Differentiation between current hepatitis infection and previous infection.	Expensive contamination of the samples examined

**Table 2:** Polymers of graphene derivatives and graphene and their usage [76].

Graphene Derivatives/Graphene	Polymer	Usage	Outcomes	Ref.
Graphene oxide	3,4-PEDOT	Bioimplants/ Biosensors	Graphene doping increases the active surface of the electrode and improves its mechanical properties. It is an excellent material in the electrode–tissue interface with a high charge injection limit for biocompatible electrical stimulation, low impedance, and high charge storage capacity.	[77]
Graphene oxide	Chitosan	DDS	GO is involved in percutaneous therapy, drug loading, and enhancing the mechanical properties of composite films. Micro-needles could penetrate the epidermis, deliver drugs, and resist insertion.	[78]
Graphene oxide nanosheets	Gelatin/polyacrylic acid	Tissue engineering	Graphene amplified the gelatin hydrogel/polyacrylic acid matrix, improving the mechanical traits (elongation at break and improved tensile strength, by 26% and 71%, respectively).	[79]
Graphene oxide	PEI	Gene delivery	GO-bonded polyethylene imide as a potential vector of DNA localization in the nucleus increases gene delivery and gene transfer.	[80]
GO/rGO	PEG	DDS	rGO has a 3–4-fold increase in light absorption compared to GO in the NIR region. Proper surface coverage and minimal size increase rGO-PEG on GO-PEG circulation time.	[81]
Graphene oxide NPs	Alginate/PEG	DDS	PEG alginate functionalizes and produces three-dimensional GONPs to deliver doxorubicin by releasing a glutathione-mediated drug.	[76]

**Table 3:** Different biomedical applications of CNTs [102].

Imaging and Diagnosis	
Biosensing	CNTs are attractive candidates for electrochemical and optical sensors because they exhibit specific electronic, optical, and mechanical properties.
Bioimaging	CNTs have many imaging applications because they have no cooling and light stability.
Remedy usage	
Thermal-phototherapy	Heat generation from near-infrared (NIR) radiation is one of the well-known capabilities of CNTs.
Tissue engineering	CNTs are significant substances for tissue engineering due to their ability to form strong 3D architectures, proliferation of cells, stimulate adhesion, mimicking of natural tissue nanofibers, rigidity, and biocompatibility.
Gene/drug delivery	CNTs carry gene/drug transmissions because of their unique needle shapes, such as immunosuppression, multifunctional surface chemistry, and high surface area.
LOC device	Small volumes of fluid flow through various channels of LOC devices for purposes such as disease models, drug screening, and cell growth. For this reason, CNTs are used as channel walls, membrane channels, and sensors in LOC devices.

**Table 4:** Summary of carbon nanostructure-based nano-biosensors for strain-specific viral detection.

Type of Nanostructure	Method of Detection	Target	Recognition Element	Linear Range	LOD	Ref.
Multi-horseradish peroxidase DNA-coated carboxyl multi-walled carbon nanotubes	Electrochemical	HCV core antigen	HCV core antibody	0.25–300 pg/mL	0.01 pg/mL	[137]
Hyaluronic acid–carbon nanotube hybrid film	Electrochemical	Anti-HBc	HBc antigen	1–6 ng/mL	0.03 ng/mL	[108]
graphene oxide (GO)/Fe_3_O_4_/Prussian blue (PB) nanocomposite	Electrochemical	HBV antigen	Hepatitis B surface antibody	0.5 pg/mL to 200 ng/mL	0.166 pg/mL	[138]
Reduced graphene oxide nanosheets	Optical	HCV cytoplasmic RNA	HCV complementary RNA	ND *	10 fM	[139]
Graphene oxide-polypyrrole	Electrochemical	HCV antigen	HCV antibody	2 to 14 ng/mL	1.63 ng/mL	[140]
Graphene quantum dots and gold-embedded polyaniline nanowires	Electrochemical	HEV	HEV antibody-conjugated polyaniline chain length	10^2^–10^7^ copies/mL	96.7 copies/mL	[141]
GQDs-SH/Ag	Electrochemical	Hepatitis C virus	Hepatitis C virus core antigen	0.05 pg/mL to 60 ng/mL	3 fg/mL	[142]
DNAzyme/nGO complex system	Optical	HCV	HCV mRNA	ND	ND	[143]
Magnetic reduced graphene oxide-copper nanocomposite	Electrochemical	HCV DNA for 1b and 6 k subtypes of HCV	o-phenylenediamine	0.5–10 nM/mL	405.0 pM/mL	[144]
Cucurbit uril (CB (7)) graphene nanocomposite	Electrochemical	HCV DNA	Cucurbit (7) uril (CB (7))	0.2–10 nmol/L	0.2 nM/mL	[145]
Graphene oxide/ferrocene-chitosan nanocomposite	Electrochemical	HBsAg	Anti-HB antibody	0.1 ng/mL to 350 ng/mL	0.1 ng/mL	[84]
rGO/AuNPs	Electrochemical	Hepatitis B virus core antigen	anti-HBcAg	3.91 ng/mL to 125.00 ng/mL	3.80 ng/mL	[40]
GQDs	Electrochemical	HBV DNA	Complementary probe HBV DNA	10 nM/mL to 500 nM/mL	1 nM/mL	[146]

* ND: not defined.

## Data Availability

All data generated or analyzed during this study are included in this published article.
